# Development and optical characterization of a multilayered head phantom for optical brain monitoring

**DOI:** 10.1117/1.JBO.31.9.095003

**Published:** 2026-09-18

**Authors:** Karina Awad-Pérez, Maria Roldan, Panicos A. Kyriacou

**Affiliations:** a City St George’s University of London, Research Centre for Biomedical Engineering, London, United Kingdom; b Crainio Limited, London, United Kingdom

**Keywords:** head phantom, cerebral photoplethysmography, extracranial circulation, skin pigmentation, optical characterization

## Abstract

**Significance:**

Optical techniques are promising tools for non-invasive cerebral monitoring; however, optical measurements acquired from the forehead can be influenced by extracranial tissues and skin pigmentation, potentially complicating interpretation of cerebral signals. Tissue-mimicking phantoms provide controlled platforms for studying these effects; however, existing head phantoms employ simplified geometries and lack the features required for cerebral photoplethysmography (PPG) investigations.

**Aim:**

We present the development and characterization of a multilayer head phantom reproducing key anatomical, optical, and hemodynamic features relevant to optical brain monitoring while enabling future investigation of confounding factors.

**Approach:**

The phantom incorporates a custom-made silicone brain with embedded vessels, a three-dimensional-printed skull, a vascularized scalp, and interchangeable skin layers representing different pigmentation levels, integrated with an *in vitro* cardiovascular system enabling selective perfusion of cerebral and extracranial compartments. The optical and colorimetric properties of the tissue-mimicking layers were assessed.

**Results:**

The scalp and skin layers exhibited absorption and reduced scattering coefficients within ranges reported for human tissues. Colorimetric assessment confirmed physiologically relevant pigmentation characteristics across the skin layers. Using the pale skin, the phantom generated measurable PPG signals across multiple wavelengths and source–detector separations.

**Conclusions:**

The multilayer head phantom provides an anatomically and optically representative platform for optical brain monitoring research. Its interchangeable skin layers and independently perfusable cerebral and extracranial compartments enable future controlled investigations of skin pigmentation and extracranial hemodynamics as confounding factors in cerebral optical measurements.

## 1 Introduction

Cerebral hemodynamic monitoring is relevant across a range of clinical applications, including traumatic brain injury, stroke, and perioperative care. Key parameters of interest include cerebral blood flow, intracranial pressure (ICP), and cerebral oxygenation. Although conventional monitoring techniques are often invasive, optical brain monitoring methods have emerged as attractive alternatives due to their non-invasive, portable, and affordable nature.^1^ Techniques such as near-infrared spectroscopy (NIRS), diffuse correlation spectroscopy, and cerebral photoplethysmography (PPG) enable real-time insights into cerebral physiological parameters. In particular, recent studies suggest that PPG signals acquired from the forehead may contain information related to ICP,^2^^–^^5^ positioning cerebral PPG as a promising approach for continuous brain monitoring.

Despite their potential, optical signals measured at the forehead are influenced not only by cerebral tissues but also by extracranial layers.^6^^–^^8^ For example, previous studies have shown that optical techniques are susceptible to signal contamination from blood flow in the scalp.^9^ Skin pigmentation has also been identified as a potential source of bias in optical measurements, with studies reporting correlations between device readings and pigmentation levels.^10^^–^^12^

Investigating confounding factors in optical brain monitoring *in vivo* is inherently challenging because individual physiological variables cannot be isolated under controlled conditions. Changes between and within subjects may all influence the measured optical signal simultaneously. As a result, it is difficult to systematically evaluate the contribution of specific factors, such as extracranial circulation or skin pigmentation, using *in vivo* experiments alone. Tissue-mimicking phantoms provide a valuable complementary approach by enabling controlled replication of anatomical structures, optical properties, and perfusion conditions.

However, many existing phantom models rely on simplified geometries, often representing the head as liquid-filled containers or rectangular tissue slabs.^13^ Anatomical layers are also frequently oversimplified or omitted, with some models excluding structures such as the scalp or skull or combining tissues with distinct optical properties into a single layer.^13^ Moreover, most phantom platforms have been developed primarily for NIRS studies and focus on DC optical signals. Consequently, multilayer head phantoms capable of producing pulsatile signals suitable for investigating cerebral PPG systems remain largely absent.^13^

This work presents the development and evaluation of a multilayer head phantom designed to replicate key anatomical, optical, and hemodynamic characteristics of the human head. The phantom incorporates vascularized brain and scalp, a cardiovascular circulation system enabling controlled perfusion of each layer, and interchangeable skin layers representing different pigmentation levels. The optical and colorimetric properties of the fabricated tissues were characterized and compared with reported values for human tissues. Finally, the functionality of the system was demonstrated through acquisition of cerebral PPG signals.

## 2 Materials and Methods

### 2.1 Brain Phantom

The brain phantom was developed following the casting methodology proposed by Roldan and Kyriacou.^14^ This approach involves the fabrication of a reusable mold, which is subsequently used to cast a silicone brain phantom with anatomically realistic geometry.

A commercially available human brain model (C20 [1017868]) manufactured by 3B Scientific (Hamburg, Germany) was used to create the mold. The model has an approximate volume of $1250\;{\mathrm{cm}}^{3}$, consistent with the reported average adult male brain volume.^15^

The brain model was suspended centrally within a Perspex box using a universal clamp mounted on a laboratory stand. The mold material consisted of silicone rubber (Polycraft GP-3481F RTV) mixed with its corresponding catalyst at a 10:1 ratio by weight. The components were manually mixed using a wooden stick and then placed in a vacuum chamber for 5 min to remove air bubbles. The final mold weighed ∼2.4  kg; therefore, the silicone mixture was prepared in batches of ∼170  g to facilitate handling and mixing.

Prior to pouring the silicone rubber mix, a silicone-based demolding agent (Ambersil heavy-duty non-silicone release) was applied to the internal surfaces of the Perspex box and to the brain model to help with separation after curing. The silicone rubber mix was left to cure overnight under ambient conditions.

**Fig. 1:**
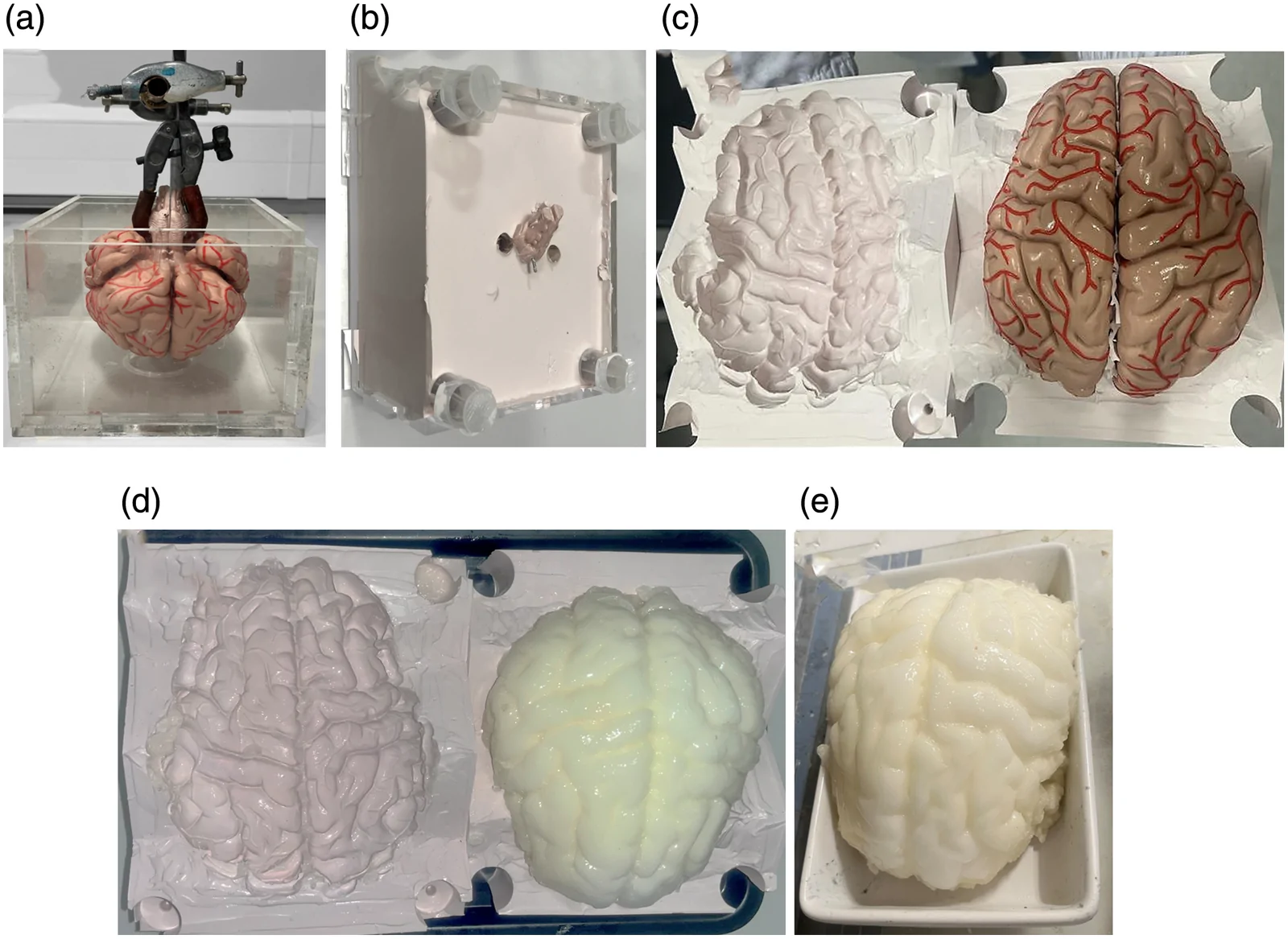
Brain fabrication process. (a) Commercial human brain model suspended within a Perspex box prior to casting. (b) Silicone mold following curing, enclosing the brain model. (c) Two mold halves with transverse zigzag cut, showing the model inside. (d) Cast brain phantom within the silicone mold. (e) Demolded brain phantom.

Once cured, the mold was carefully cut transversely in a zigzag pattern. The zigzag allowed for precise realignment of the mold halves in the following steps of the phantom fabrication process. The transverse cut also allowed the top half of the mold to press down during casting, preventing silicone leakage and preserving the phantom’s structural integrity. The brain mold development process is shown in Fig. 1.

The brain phantom was fabricated using Sylgard 527 (DOWSIL™, Dow Europe GmbH, Wiesbaden, Germany) in a 1:1 A:B ratio, chosen to replicate the mechanical properties of brain tissue.^14^ Titanium dioxide (${\mathrm{TiO}}_{2}$) particles with a primary crystal size of 550 nm (Altiris 550, Venator Corporation, Teesside, United Kingdom) were incorporated into the material to closely match optical properties reported in the literature.^14^

Part A of Sylgard 527 was first added to a mixing container, followed by the addition of ${\mathrm{TiO}}_{2}$ at a concentration of 0.1 g ${\mathrm{TiO}}_{2}$ per 97 g of Sylgard 527. The mixture was blended using an electric stirrer for ∼4  min to ensure uniform dispersion of the particles. Part B was then added, and the mixture was blended for an additional 4 min. To remove air bubbles, the mixture was placed under vacuum twice, each time for 10 min. As with the mold fabrication, the silicone mixture was prepared in smaller batches of ∼300  g to facilitate thorough mixing. The developed brain phantom had a total weight of 840 g [Fig. 1(e)].

Prior to pouring the brain mixture in the mold, silicone demolding agent (Ambersil heavy-duty silicone release) was applied to it. The prepared mixture was then poured into the molds, which were wrapped in stretch film wrap to prevent leakage and left overnight to allow for initial precuring. Curing was carried out in an oven at 125°C for 5 h. The brain was then demolded, and a final curing step was performed at 125°C for an additional hour to complete the process.

### 2.2 Brain Vasculature

To assess cerebral PPG, the brain phantom had embedded vasculature capable of replicating the pulsatile variations that occur in blood vessels during the cardiac cycle. Cerebral PPG sensors are typically positioned on the forehead and interrogate the frontal lobe. Forehead placement is also standard in other optical techniques, such as NIRS. For this reason, a representation of the anterior cerebral arteries (ACAs) was incorporated into the phantom design.

To simulate the ACAs, three custom-made silicone vessels with an internal diameter of 2.6 mm were fabricated and embedded within the brain phantom. This diameter falls within the range reported by Mujagić (0.7 to 3.7 mm).^16^ The vessels were developed using a continuous dip-coating process as described by Nomoni et al.,^17^ a technique proven effective in fabricating distensible vessels for PPG signal acquisition.^17^ The commercial tubing used had a 0.3-mm wall thickness and a 2.0-mm internal diameter, resulting in a final lumen diameter of 2.6 mm (inner diameter+2× wall thickness=2.0  mm+2×0.3  mm=2.6  mm). The silicone mixture was prepared using polydimethylsiloxane, specifically Sylgard™ 184 (DOWSIL™, Dow Europe GmbH, Wiesbaden, Germany), by combining 25 g of elastomer base with 10% catalyst (2.5 g). The mixture was degassed in a vacuum chamber twice for 3 min to remove air bubbles and then allowed to precure at room temperature for 3 h. Final curing was completed using a dip-coater. Once cured, the custom vessels were gently peeled from the commercial tubing and tested for leaks to ensure no pores were present.

An additional vessel representing the middle cerebral artery (MCA) was also embedded within the brain phantom to complete the circulation pathway, allowing the fluid to exit at the posterior region of the head. The MCA was represented by a commercial silicone vessel with a 3.2-mm diameter.

The ACA vessels were positioned ∼3  mm beneath the frontal lobe surface, and the MCA vessel was embedded at a depth of 3 mm beneath the temporal lobe surface. For embedding, a cylindrical copper rod was used as a guide. The vessel was first placed over the copper rod, which was pre-coated with a silicone-based demolding agent (Ambersil heavy-duty silicone release) to facilitate later removal. The rod, covered with the vessel, was then inserted into the brain phantom and carefully removed, leaving the vessel embedded in place without deformation or damage.

Custom-made connectors were three-dimensional (3D) printed in clear photopolymer resin (Clear Resin V4; Formlabs, Somerville, Massachusetts, United States). These connectors were used to merge the three ACA vessels located in the frontal lobe into a single inlet, which exited the skull to connect with the external circulation system.

### 2.3 Skull Phantom

The skull phantom was designed using a pre-existing computational skull model, which was modified using Fusion 360 (Autodesk Inc., San Rafael, California, United Sates), and consisted of two anatomical components: the calvaria and the maxilla. The model was scaled such that the calvaria and maxilla fitted precisely together and were anatomically consistent with the fabricated brain phantom.

**Fig. 2:**
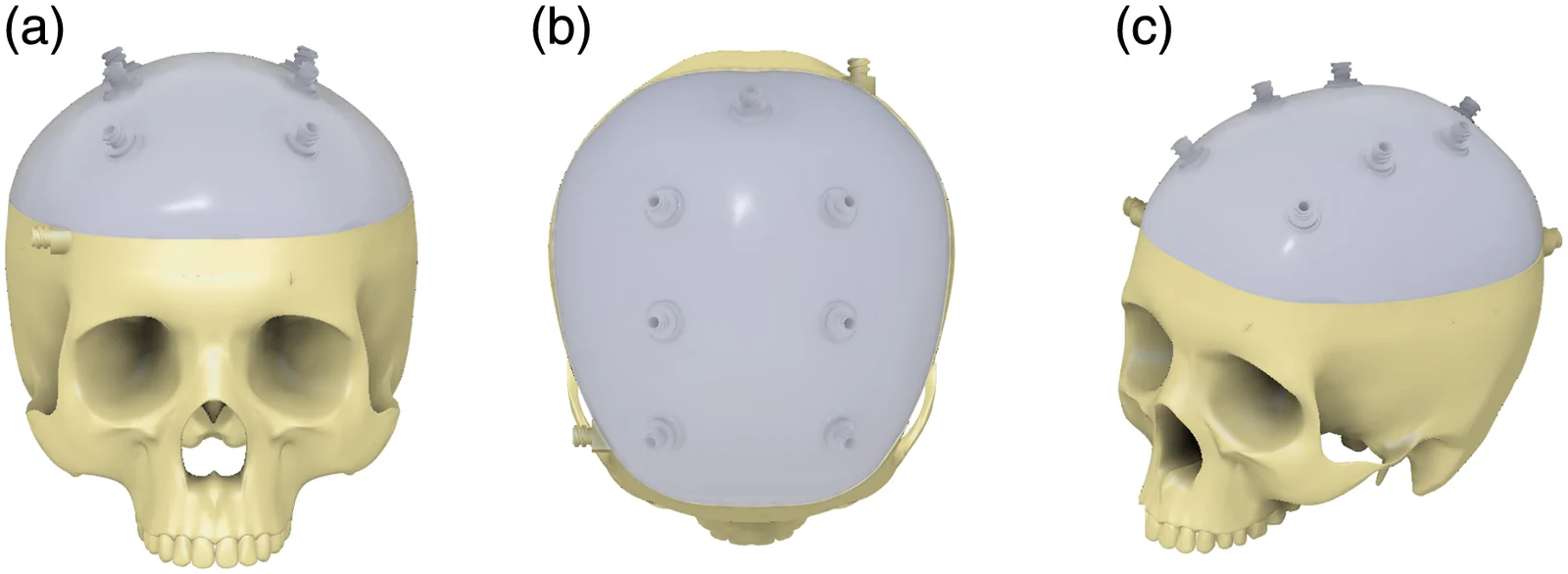
Skull phantom design. (a) Frontal view. (b) Top view. (c) Isometric view. The calvaria is shown in gray and the maxilla in yellow.

The overall skull thickness was set to 5.2 mm, which lies within the range reported for adult human skull thickness (4.7 to 14.7 mm) by Thulung et al.^18^ Luer connector outlets were incorporated into the design of the maxilla to facilitate standardized connections between the cardiovascular system and the embedded cerebral arteries. Additional Luer ports were integrated into the calvaria to enable connection to the cerebrospinal fluid (CSF) circulation mechanism and to allow ICP monitoring. A schematic of the skull design is shown in Fig. 2.

The calvaria and maxilla were 3D printed in clear resin (Clear Resin V5; Formlabs, Somerville, Massachusetts, Unitesd States). After printing, support structures were manually removed, and the parts were post-cured in a ultraviolet curing chamber at 60°C for 20 min.

The calvaria and maxilla were mechanically joined using a custom clamping system that applied uniform downward pressure to hold both components together.^14^ To ensure a fluid-tight seal between the two parts, a silicone sealing ring was fabricated using silicone (Gel 00; Polytek Development Corp., Easton, Pennsylvania, United States). Part A of the silicone was mixed with 2.5% retarder and combined with part B in a 1:1 ratio to create the seal. The sealing capability of the system was evaluated by filling the cranial cavity with water and inserting a pressure sensor (single-use pressure sensor; PendoTECH, Township, New Jersey, United States) into one of the calvaria Luer ports. ICP was increased incrementally up to 25 mmHg using a syringe, while pressure was continuously monitored. Visual inspection confirmed the integrity of the seal, with no observable fluid leakage.

Following verification of the seal, a CSF circulation mechanism was implemented to allow controlled adjustment of ICP. This system consisted of a syringe filled with water, which served as a CSF analog, connected to a programmable pump (Legato 180, KD Scientific Inc., Holliston, Massachusetts, United States). By gradually increasing the water volume within the cranial cavity, ICP could be increased in a controlled and repeatable manner. Changes in ICP were continuously monitored using the pressure sensor located in the calvaria.

### 2.4 Scalp Layer

To evaluate the effect of extracerebral layers on the PPG signal, a scalp phantom was fabricated. This phantom was designed to replicate forehead tissue and its circulation, accurately reproducing real tissue thickness, vessel diameter, anatomical structure, and optical properties.

**Fig. 3:**
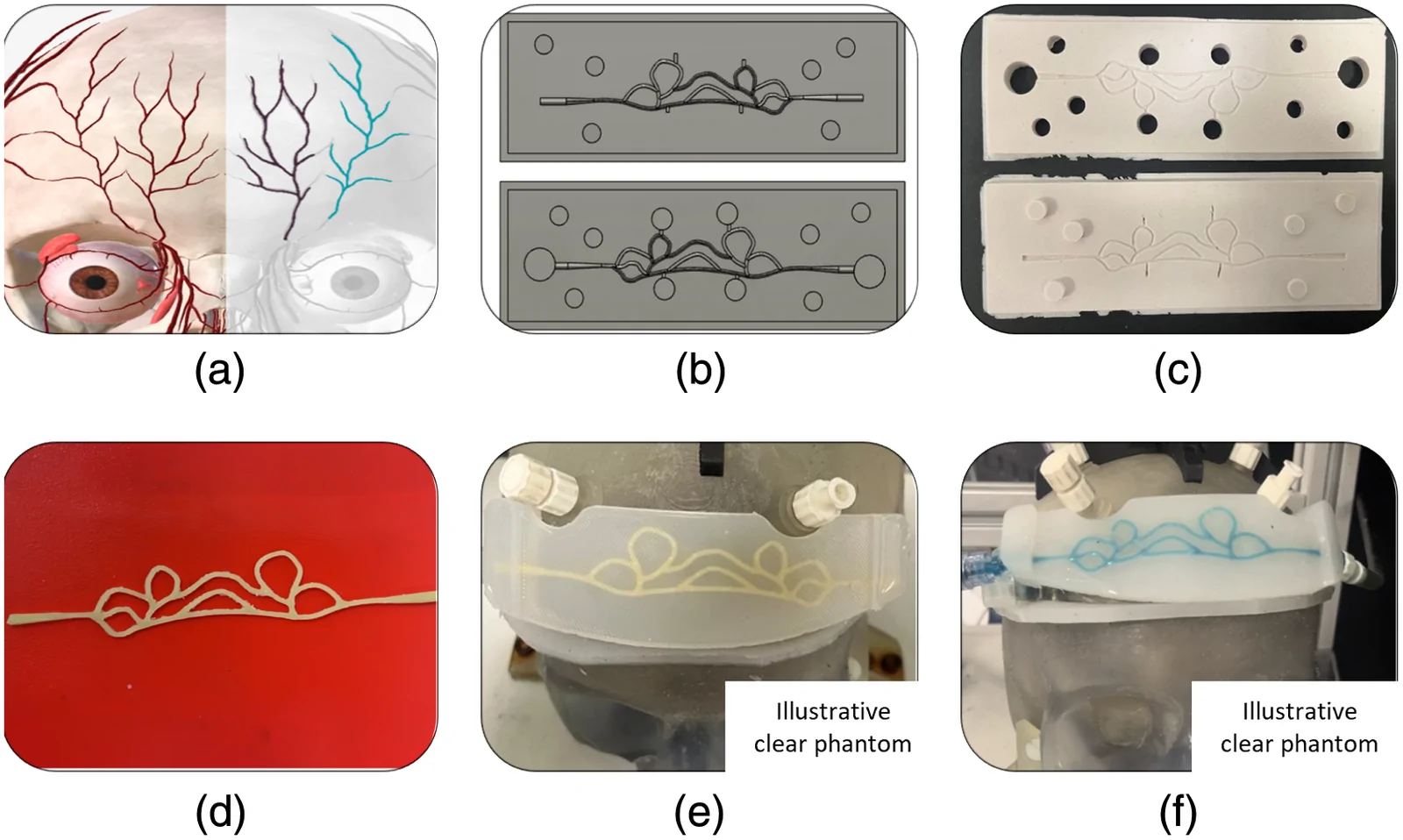
Summary of the lost-wax fabrication technique used to produce the scalp tissue phantom. (a) Trace vascular network model of the forehead circulation. (b) Design and 3D printing of the two-part negative mold. (c) Fabrication of the flexible silicone mold. (d) Creation of the vascular network using a meltable material (wax). (e) Embedding of the wax network within the tissue-mimicking material. (f) Removal of the wax to reveal the final channel-based phantom. The actual scalp phantom used in the experiments was optically characterized; however, a clear silicone phantom is shown here for illustrative purposes to visualize the embedded channels.

The phantom was developed using a lost-wax technique, in which a meltable network is embedded within the tissue and then melted, creating hollow channels. A similar approach was employed by Nagassa et al.^19^ to fabricate cerebral artery aneurysm phantoms for neurosurgical simulation. The fabrication process consisted of several steps, which are described in detail below and summarized in Fig. 3.

**Fig. 4:**
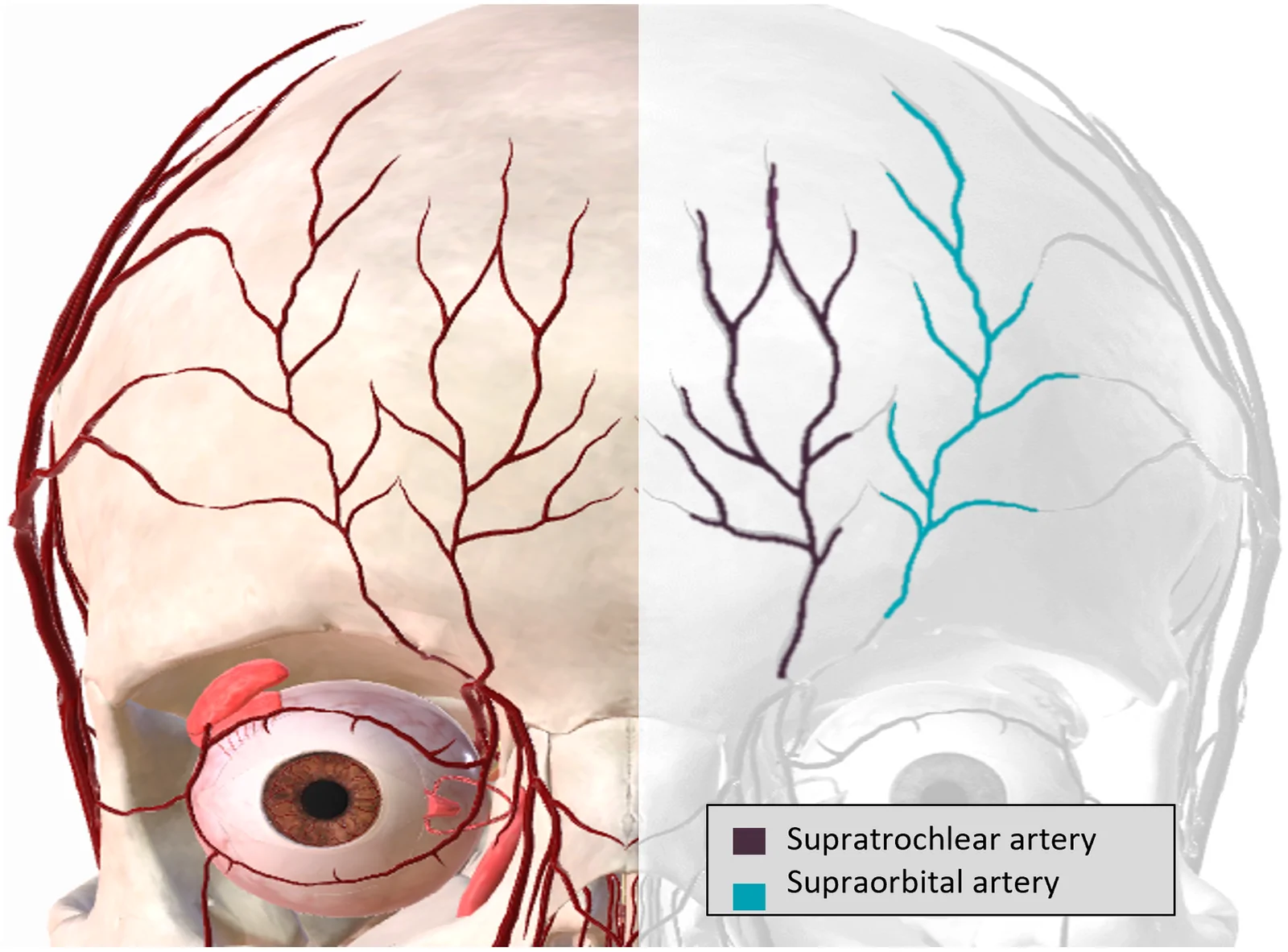
Model of extracranial circulation. Anatomical representation of the supratrochlear and supraorbital arteries used as a reference for the design of the vascular network. Modified from Ref. 20.

The first step was replicating the branching patterns and anastomoses of the human forehead vessels. To do so, a model representing the major forehead arteries was downloaded from Ref. 20 and traced (Fig. 4). The resulting network consisted of the anastomoses of the supratrochlear and supraorbital arteries. This network was traced in Fusion 360 (Autodesk Inc., San Rafael, California, United States) and scaled to match real human dimensions. The network’s diameter was set to 1.2 mm, within the range reported in the literature,^21^ and the total length was adjusted to 11.5 cm to align with the head phantom dimensions. The diameter of both ends of the network was slightly larger (2.5 mm) to allow connection using standard Luer connectors.

**Fig. 5:**
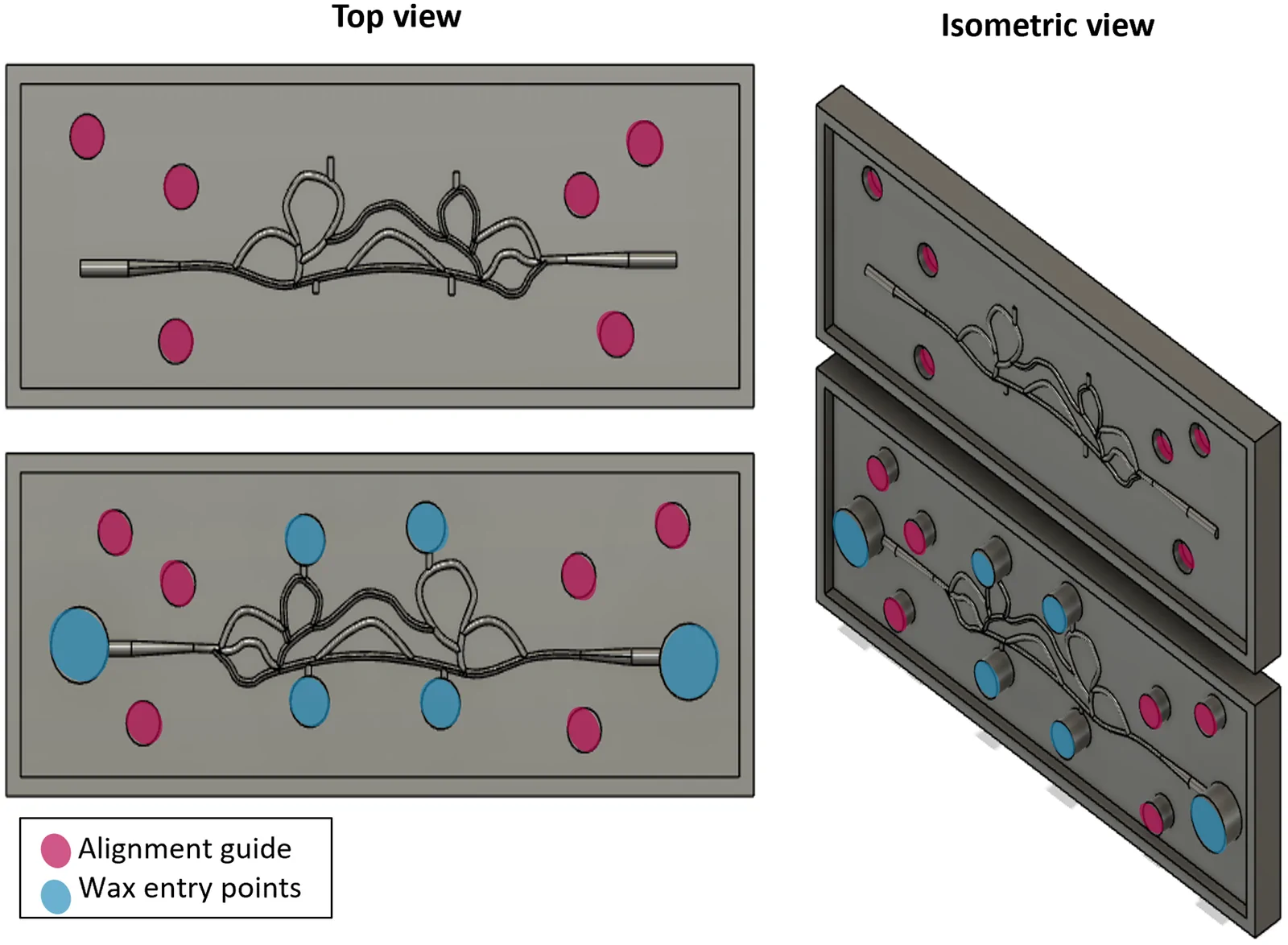
Two-part negative mold CAD design for fabrication of the extracranial vascular network. Top and isometric views of the two-part negative mold showing the embedded vascular geometry. Colored markers indicate alignment guides (magenta) and wax entry points (blue) used during the lost-wax fabrication process.

**Fig. 6:**
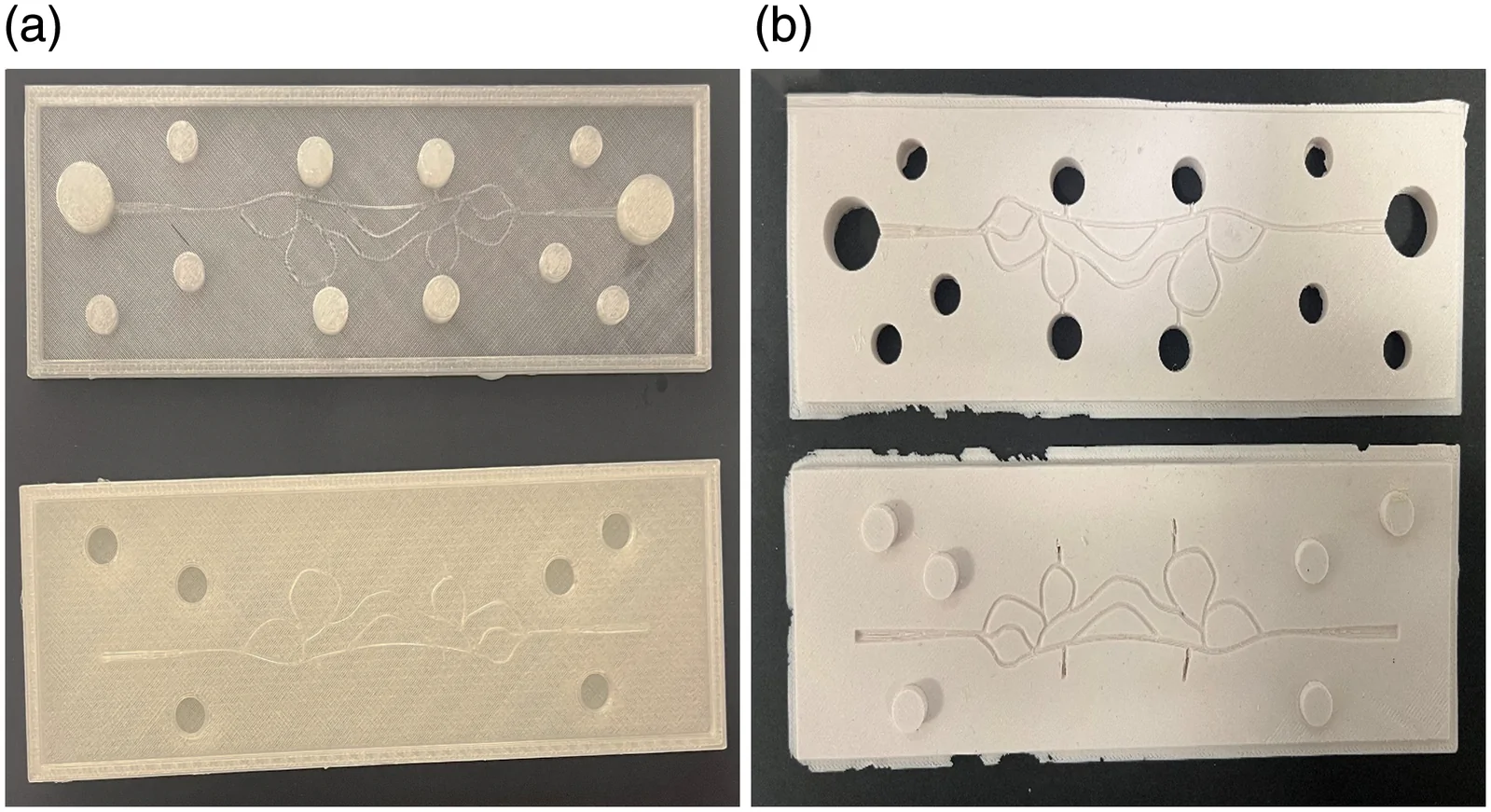
Molds used for fabrication of the extracranial vascular network. (a) Two-part negative mold fabricated in PLA. (b) Flexible Polycraft mold cast from the PLA negative.

Based on the vascular network, a two-part negative mold was designed using Fusion 360 (Autodesk Inc., San Rafael, California, United States) (Fig. 5). The design included guide cylinders to ensure accurate alignment between the two mold halves, as well as six wax entry points. Due to the length and complexity of the vascular network, more than two entry points were necessary to ensure that the melted wax could reach all regions of the mold uniformly and without obstruction. The two-part negative mold was printed in polylactic acid (PLA) using an Original Prusa i3 MK3S+ (Prusa Research by Josef Prusa, Prague, Czech Republic) [Fig. 6(a)].

A flexible mold [Fig. 6(b)] was fabricated using the negative templates and Polycraft GP-3481F RTV silicone, combined with its catalyst in a 10:1 ratio. The mixture was degassed in a vacuum chamber for 3 min to eliminate air bubbles then poured into the negatives and left to cure overnight under ambient conditions.

Once the molds were cured, the next step involved casting the vascular network using a meltable material. Beeswax was melted in a water bath until it was liquid. To prevent solidification, each part of the silicone mold was preheated in an oven prior to wax injection. The two halves of the mold were then aligned using the guiding cylinders, and the wax was poured through the inlets.

A scalp tissue phantom mold was designed and printed with a thickness of 4 mm to match reported human scalp dimensions.^22^ The vessel network was placed approximately in the middle, resulting in a distance of around 1.4 mm from the channel to the top of the phantom, where the optical sensors are placed. A thicker layer of 13 mm was designed for the ends of the phantom, providing stability and preventing ruptures where the connectors are attached.

The tissue-mimicking material was created using Gel 00 silicone (Polytek Development Corp., Easton, Pennsylvania, United States) as the base, with ${\mathrm{TiO}}_{2}$ (Kronos 2190, Kronos Titan GmbH, Leverkusen, Germany) serving as the optical scatterer and Bismarck brown (BDH Chemicals Ltd., London, England) as the absorber. A Bismarck brown stock solution was prepared at a concentration of 25 mg/mL using isopropanol as the solvent. The final mixture included 7.74  μL of Bismarck brown stock solution per gram of silicone and 1 mg of ${\mathrm{TiO}}_{2}$ per gram of silicone.

The fabrication process began by mixing part A of the silicone with the Bismarck brown stock solution and ${\mathrm{TiO}}_{2}$ for ∼3  min using an electric stirrer, ensuring a homogeneous mixture with no visible particles. Once this was achieved, 1.5% retarder was added. Part B was then mixed with part A in a 1:1 ratio, and the mixture was placed under a vacuum for 3 min to remove air bubbles. After this step, the mixture was poured into the tissue box, which had the meltable network inside.

Once the tissue mix had cured, the wax was melted out of the phantom by placing it directly in an oven. A syringe with boiling water was used to flush out any remaining residue, resulting in hollow vascular channels within the phantom.

### 2.5 Skin Layers

**Fig. 7:**
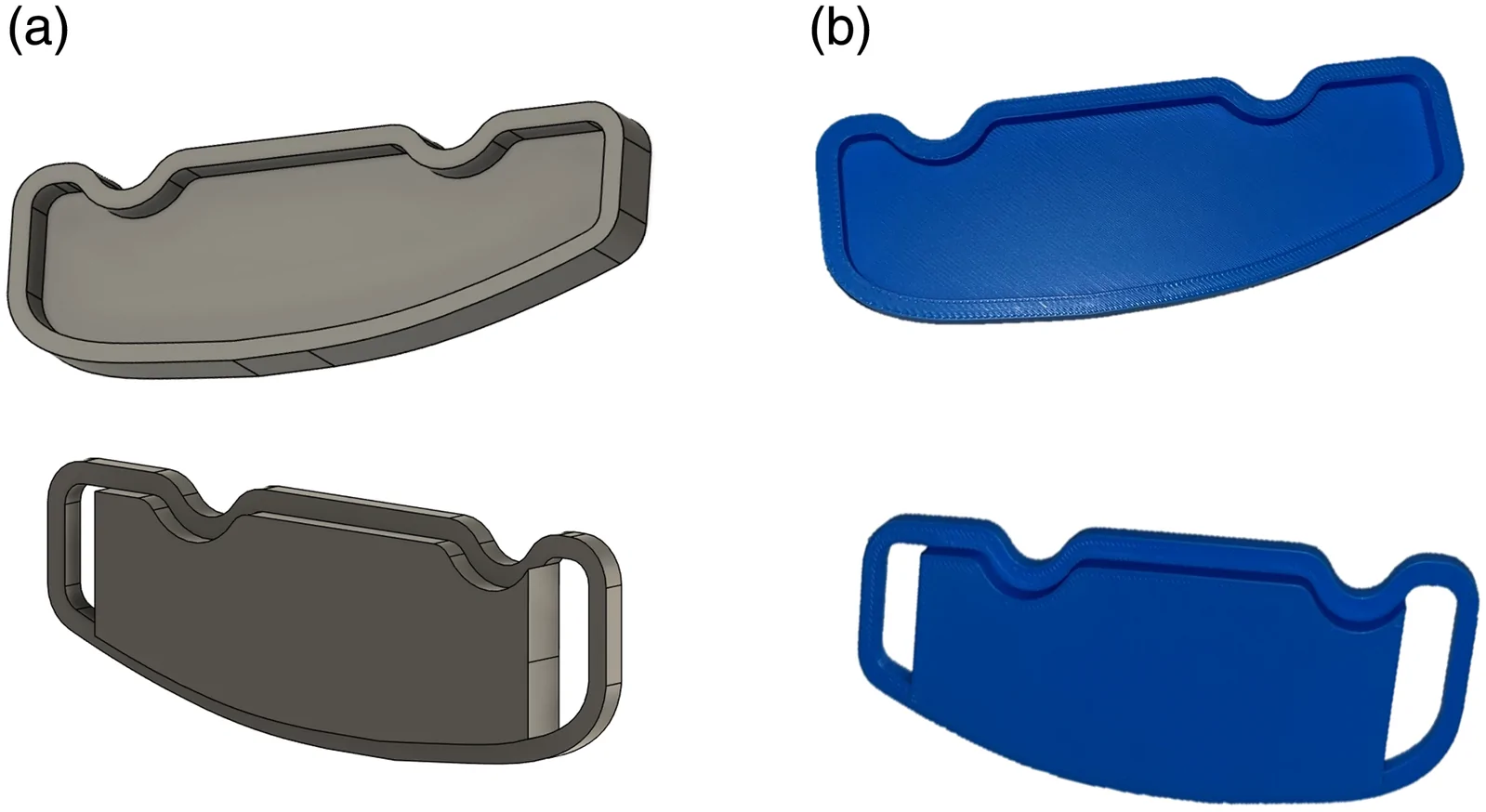
Skin phantom mold. (a) CAD design of the two-part negative mold showing the upper and lower mold components designed to produce a controlled skin thickness of 1.3 mm. (b) Corresponding 3D-printed PLA mold fabricated.

Skin phantoms representing different pigmentation levels were fabricated as interchangeable slabs that could be placed on top of the scalp phantom. A two-part negative mold (Fig. 7) was designed in Fusion 360 (Autodesk Inc., San Rafael, California, United States) and printed in PLA using an Original Prusa i3 MK3S+ (Prusa Research by Josef Prusa, Prague, Czech Republic). The mold was designed to produce slabs with a controlled thickness of 1.3 mm, corresponding to the average skin thickness reported for the central forehead region.^23^ An overflow channel was designed along the mold edges to allow excess silicone to escape and ensure a flat slab with the desired thickness. After curing, the excess material was trimmed.

The skin phantoms were fabricated using Gel-00 silicone (Polytek Development Corp., Easton, Pennsylvania, United States) as the base material. ${\mathrm{TiO}}_{2}$ particles (Kronos Titan GmbH, Leverkusen, Germany) were added as the optical scatterer at a concentration of 1 mg/g of silicone. Optical absorption was controlled using combinations of Bismarck brown, India ink, Epolight 5010, and Epolight 5532 (Epolin Inc., Newark, New Jersey, United States).

Stock solutions were prepared prior to phantom fabrication. Bismarck brown (BDH Chemicals Ltd., London, United Kingdom) was dissolved in isopropanol at 25 mg/mL. India ink (Winsor & Newton, London, United Kingdom) was diluted in deionized water to prepare 0.045% *v*/*v* and 1% *v*/*v* stock solutions, depending on the required pigmentation level. Epolight 5010 and Epolight 5532 dyes were dissolved in acetone at concentrations of 2 and 1 mg/mL, respectively. Gentle heating in a water bath was used to facilitate dye dissolution.

Three skin phantoms representing “pale,” “medium,” and “dark” pigmentation levels were fabricated by varying the concentration of the absorbing dyes. The specific compositions used for each skin phantom are summarized in Table 1.

**Table 1:** Skin phantom formulations. All components are reported relative to the total silicone mass (g), corresponding to the combined mass of parts A and B. A:B indicates the mixing ratio between silicone parts A and B. *R*, silicone retarder. ${\mathrm{TiO}}_{2}$, titanium dioxide. Bismarck, Epolight 5010, Epolight 5532, and India ink correspond to absorber stock solutions; for India ink, the concentration of the stock solution is indicated in parentheses.

Skin phantom	A:B	*R* (g/g)	${\mathrm{TiO}}_{2}\;\left(\mathrm{mg}/g\right)$	Bismarck (μL/g)	Epolight 5010 (μL/g)	Epolight 5532 (μL/g)	India Ink (% *v*/*v*)
Pale	1:1	0.1	1	7.2	76	—	53 μL/g (0.045%)
Medium	1:1	—	1	40	—	100	20 μL/g (1%)
Dark	1:1	—	1	100	—	200	30 μL/g (1%)

During fabrication, the required amount of part A silicone was first mixed with the ${\mathrm{TiO}}_{2}$ scatterer and the corresponding absorber stock solutions. The mixture was blended using an electric stirrer until a homogeneous dispersion was achieved. When required, a silicone retarder (Polytek Development Corp., Easton, Pennsylvania, United States) was added to extend the working time before curing. Part B was then added, and the mixture was degassed under vacuum to remove air bubbles. The mixture was subsequently poured into the mold and left to cure under ambient conditions.

### 2.6 Optical Characterization

The optical properties were determined to verify that the fabricated scalp and skin phantoms matched reported values for human tissue. Diffuse transmittance and diffuse reflectance spectra were obtained using a 100-mm InGaAs integrating sphere module attached to a Lambda 1050 dual-beam spectrophotometer (Perkin Elmer Corp., Waltham, Massachusetts, United States). An empty cuvette with the same characteristics as the test samples was used as a reference. The spectra were collected from 500 to 1000 nm, with a data interval of 1 nm. However, the analysis primarily focused on the 750 to 900 nm range, as this corresponds to the operating range of the optical sensor. The gain was set to 0, and the response time was set to 0.2 s. Each sample was tested in three cycles for replicates, and the results were averaged.

The absorption (μa) and reduced scattering coefficients ($\mu s^{\prime }$) were calculated from the acquired spectra using the inverse adding doubling method.^24^ This method is widely used in tissue optics and is ideal for processing spectrophotometry data obtained with integrating spheres to obtain optical coefficients.^25^ The sample thickness was set to 10 mm, corresponding to the dimensions of the plastic cuvette. The specifications of the integrating sphere were also taken into account, and the anisotropy factor (*g*) was set to 0.9, as reported in various *in vivo* and *in vitro* studies.^26^^–^^29^

Colorimetric and melanometric measurements of the skin phantoms were acquired using a Skin ColorCatch device (Delfin Technologies Ltd., Kuopio, Finland). This instrument provides measurements of melanin index, erythema index, the Individual Typology Angle (ITA°), and CIE L*a*b* color coordinates.

**Fig. 8:**
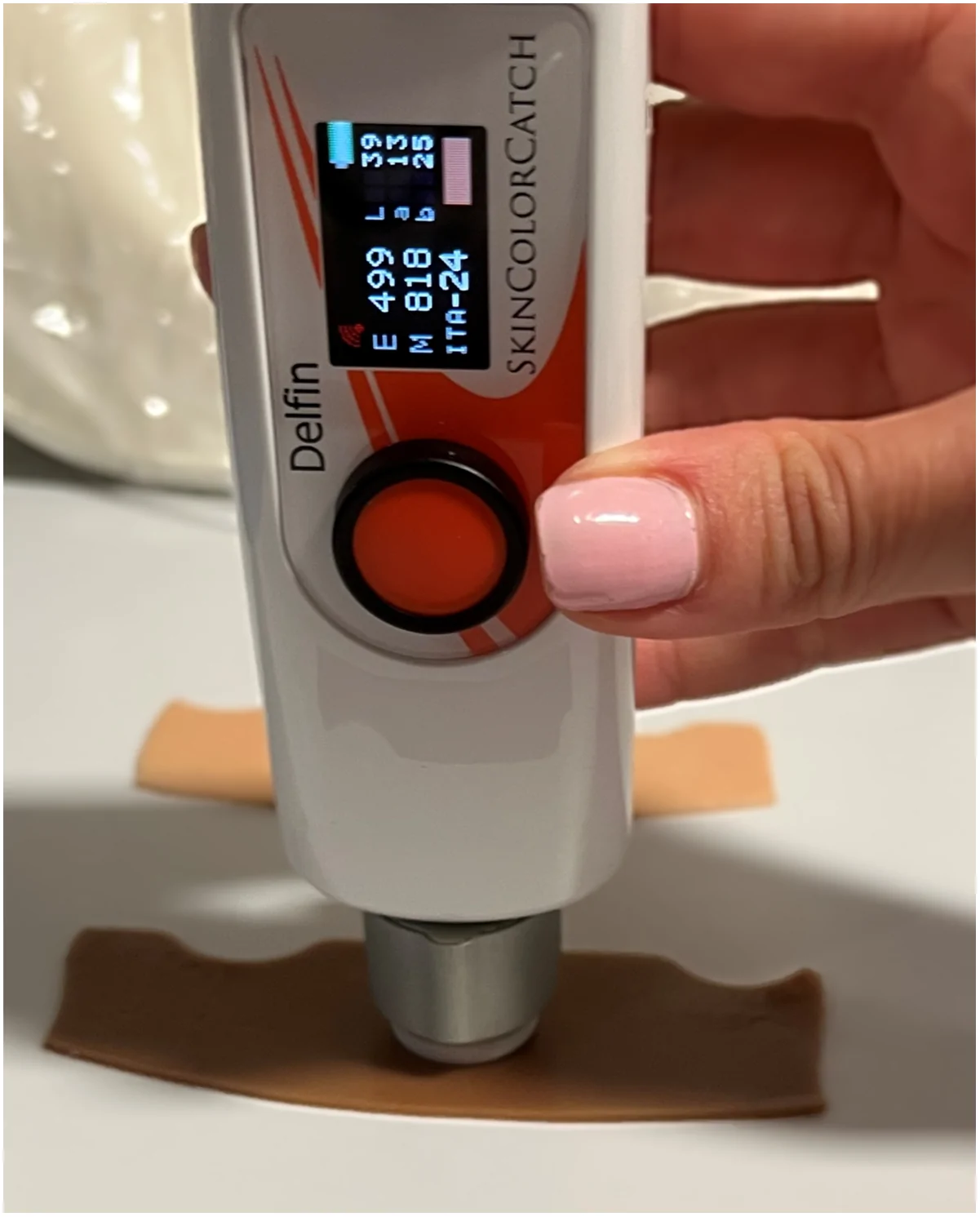
Colorimetric and melanometric measurements of skin phantom using the Skin ColorCatch device.

The CIE L*a*b* color space describes color using three parameters: L* represents lightness (0=black, 100=white), a* the red–green axis, and b* the yellow–blue axis. These parameters are commonly used to quantify skin pigmentation and erythema.^30^ Skin pigmentation was further assessed using the ITA°, a parameter used as a quantitative indicator of skin pigmentation because it correlates with melanin content.^30^^,^^31^ During measurements, the device probe was positioned perpendicular to the surface of the skin phantom as shown in Fig. 8.

### 2.7 Cardiovascular System and Phantom Evaluation

A cardiovascular closed-loop system was used to generate a pulsatile flow, simulating the dynamic action of the heart and blood vessels. A pulsatile pump (Harvard Apparatus 55-3305; Harvard Apparatus, Holliston, Massachusetts, United States) was used to replicate the heart’s pumping mechanism. Fluid circulated continuously through the closed-loop system, passing through the phantoms and arriving at a sealed venous reservoir (Affinity Pixie, Medtronic, Minneapolis, Minnesota, United States) before returning to the pump for recirculation. The reservoir filter was removed to prevent absorption of the dyes used in the blood-mimicking fluid.

**Fig. 9:**
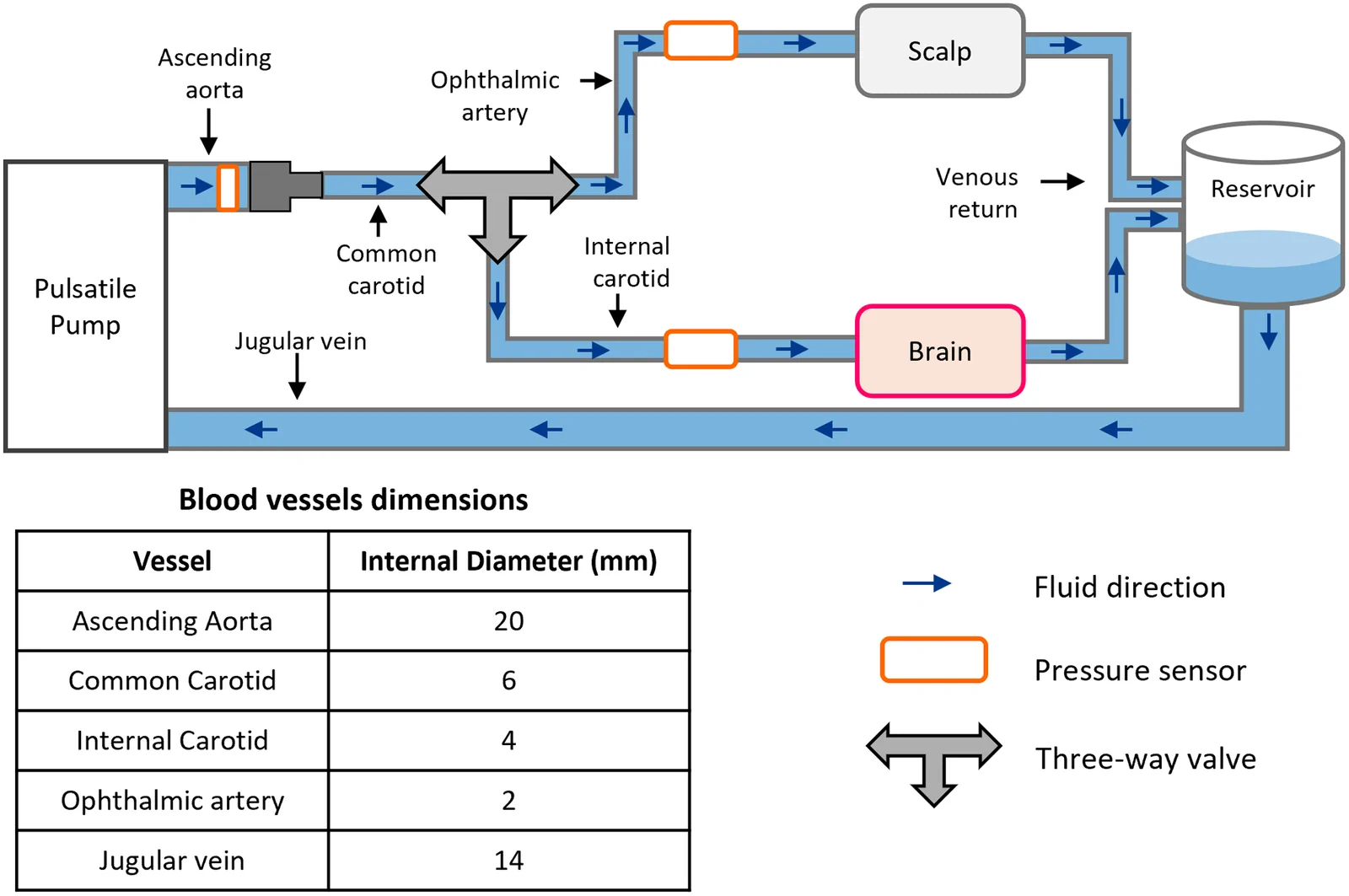
Cardiovascular system representation and blood vessels’ internal diameter. Flow is generated by a pulsatile pump and distributed to the scalp and brain phantoms via a branched pathway.

The simulated vessels within the system included the ascending aorta (inner diameter=20  mm, length=124  mm), the common carotid artery (inner diameter=6  mm, length=136  mm), the left internal carotid artery (inner diameter=4  mm, length=86  mm), the left ophthalmic artery (inner diameter=2  mm, length=86  mm), and the jugular vein (inner diameter=14  mm). Pressure sensors (single-use pressure sensor; PendoTECH, Township, New Jersey, United States) were positioned at the ascending aorta and before the scalp and brain phantom. A schematic representation of the cardiovascular system, together with a summary of vessel inner diameters, is shown in Fig. 9.

The blood-mimicking fluid consisted of a mixture of two solutions designed to replicate the optical behavior of oxygenated and deoxygenated hemoglobin, following the formulation reported by Roldan and Kyriacou.^14^ A 25:75 ratio of oxygenated to deoxygenated components was used to approximate the predominance of venous blood in the cerebral circulation,^32^ consistent with assumptions commonly implemented in optical brain oximetry systems.^33^

The assembled head phantom was integrated into the cardiovascular system to verify its ability to generate measurable cerebral PPG signals. The pulsatile pump was set to 60 beats/min, with a 40/60 systole–diastole ratio and a stroke volume of 20 cc; peripheral resistance was not adjusted in this study. The mean ICP was maintained at ∼10  mmHg. For this preliminary evaluation, only the pale skin phantom was placed on top of the scalp layer.

PPG signals were acquired using a custom optical probe and acquisition module developed by Crainio (London, United Kingdom). The probe incorporated light-emitting diodes (LEDs) operating at 770, 810, and 880 nm, and two photodetectors positioned at 1 cm (proximal) and 3 cm (distal) source–detector separations. The probe was positioned on the forehead region of the phantom. The acquisition system was configured with a sampling frequency of 200 Hz, and the drive current for each LED was set to 25 mA.

## 3 Results and Discussion

### 3.1 Head Phantom

**Fig. 10:**
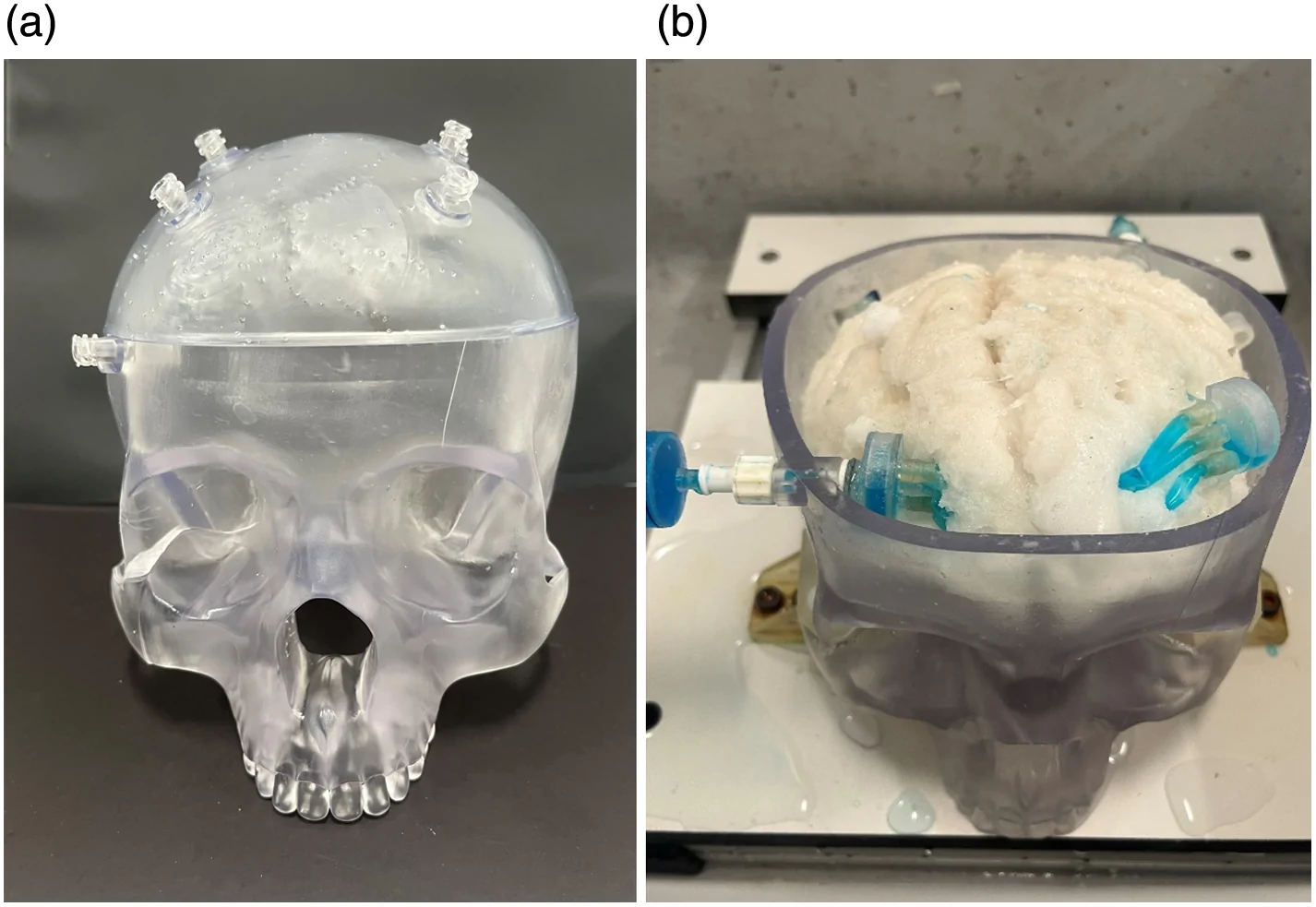
Fabricated skull and brain phantom. (a) 3D-printed skull consisting of the calvaria and maxilla with integrated Luer ports. (b) Brain phantom with embedded custom-made silicone vessels representing cerebral arteries.

The fabricated multilayer head phantom consisted of anatomically representative brain, skull, scalp, and skin designed to reproduce the anatomical and optical characteristics of the human head. The skull, fabricated using 3D printing, provided a rigid enclosure for the brain phantom and incorporated fluid ports enabling connection to the cardiovascular and CSF circulation systems [Fig. 10(a)]. The silicone brain included embedded vessels representing cerebral arteries [Fig. 10(b)].

**Fig. 11:**
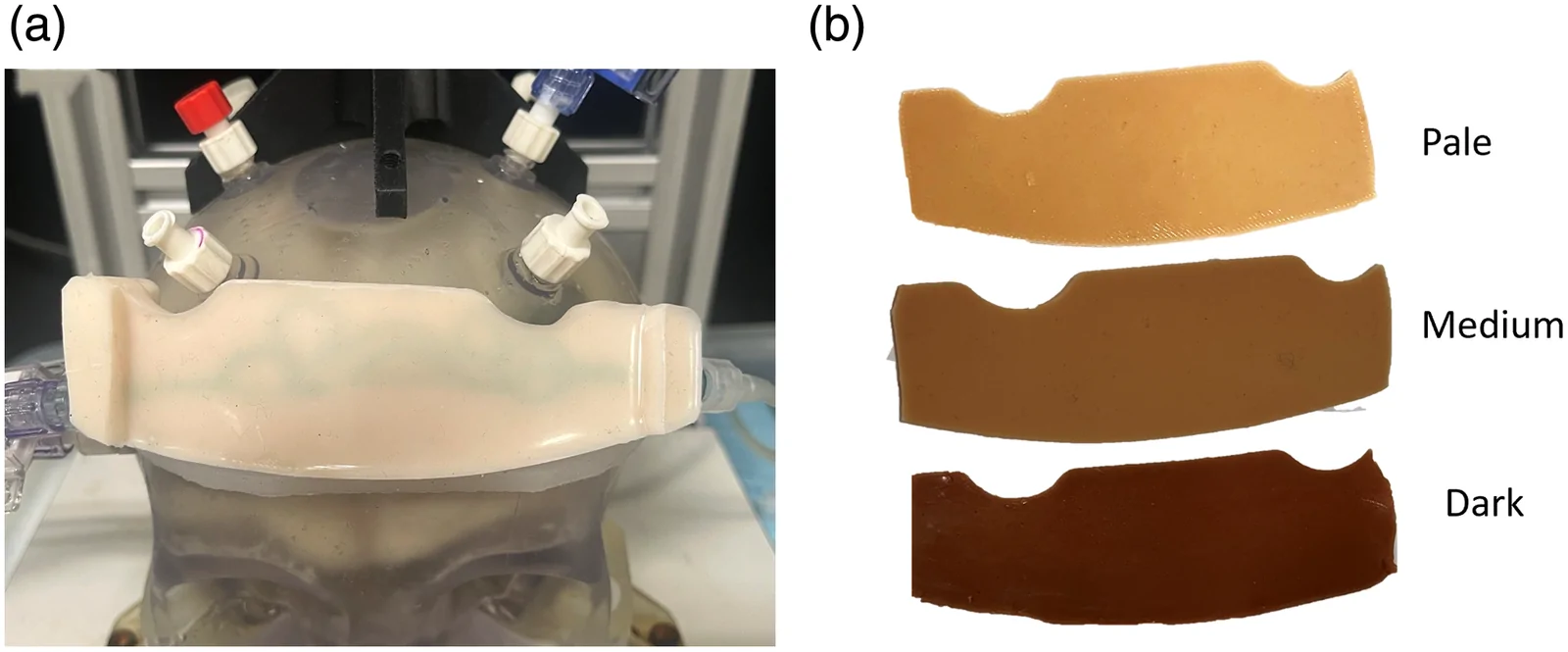
Extracranial phantom layers. (a) Vascularized scalp phantom mounted on top of the skull phantom. (b) Interchangeable skin phantoms representing different pigmentation levels (pale, medium, and dark) fabricated as thin slabs for placement above the scalp phantom.

To replicate extracranial circulation, the vascularized scalp phantom was positioned on top of the skull [Fig. 11(a)]. This layer contained a network of hollow channels representing the supratrochlear and supraorbital arteries. Interchangeable skin slabs representing three pigmentation levels (pale, medium, and dark) were fabricated to be placed directly on top of the scalp phantom [Fig. 11(b)].

**Fig. 12:**
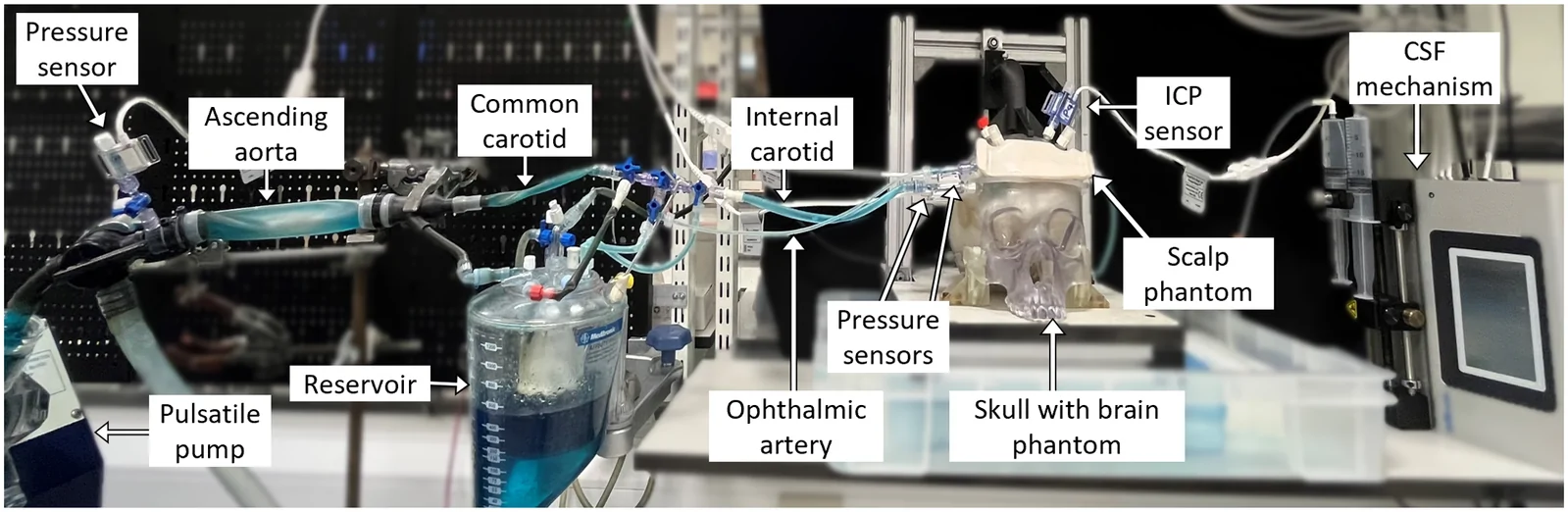
Fully assembled head phantom and cardiovascular closed-loop system. Photograph of the experimental setup including arterial pathways supplying the scalp and brain phantoms, pressure sensors, and the CSF mechanism to control ICP.

The fully assembled cardiovascular closed-loop system used in the experiments is shown in Fig. 12. The main components of the setup included the pulsatile pump, venous reservoir, arterial pathways supplying the scalp and brain phantoms, pressure sensors, and the CSF mechanism for ICP control.

### 3.2 Optical Properties

**Fig. 13:**
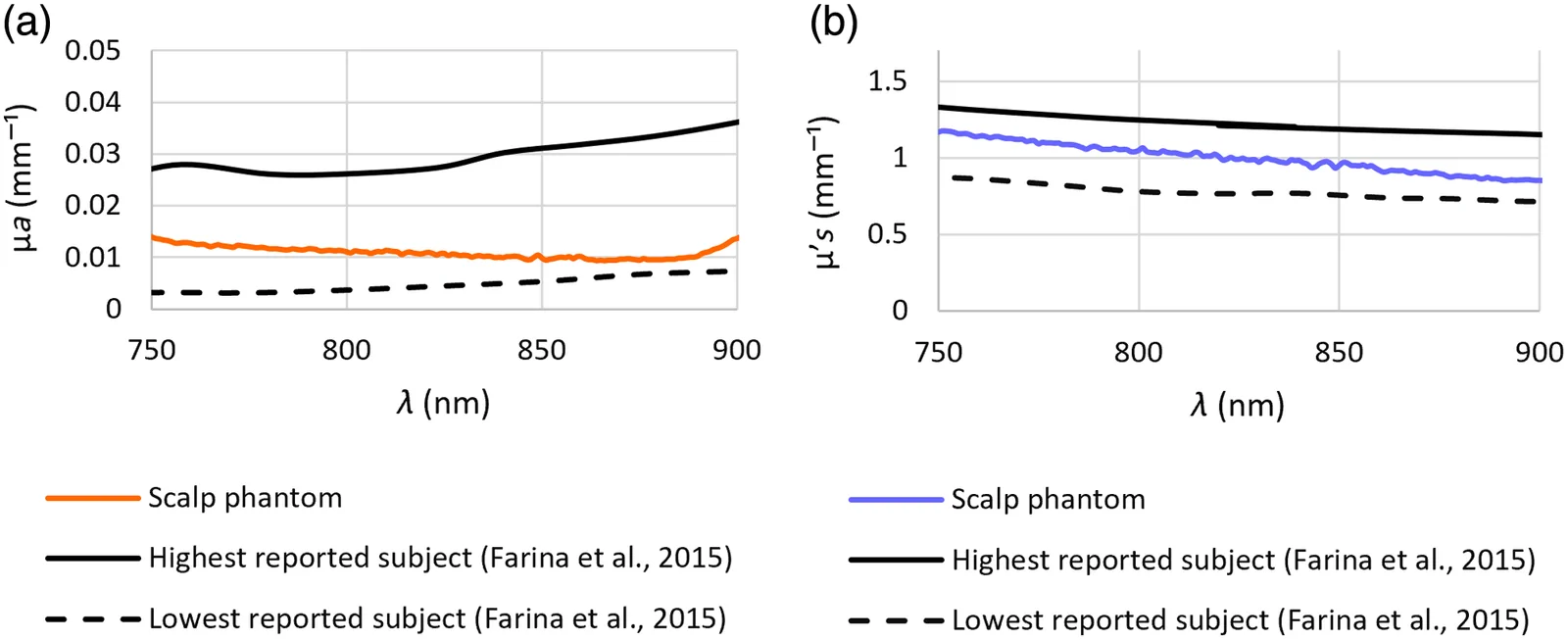
Optical properties of the scalp phantom compared with *in vivo* forehead measurements. (a) Scalp absorption coefficient (μa) and (b) scalp reduced scattering coefficients ($\mu s^{\prime }$), across the 750- to 900-nm wavelength range. The phantom coefficients are plotted alongside the lowest and highest values reported for human forehead tissue by Farina et al.

The optical properties of the brain phantom, skull, and blood-mimicking fluid were previously validated.^14^ Therefore, the optical characterization in this study focused on the fabricated scalp and skin phantoms. The μa and $\mu s^{\prime }$ were compared with values reported in the literature. For the scalp phantom, reference data were taken from a study by Farina et al.,^34^ in which *in vivo* optical properties of the forehead were measured in nine subjects. The scalp phantom values were compared against the lowest and highest reported values from that study, as plotted in Fig. 13. Across the measured wavelength range (750 to 900 nm), the coefficients of the scalp phantom fell within the range of values reported for human forehead tissue. The maximum standard deviation across the measured range was $0.0003\;{\mathrm{mm}}^{-1}$ for μa and $0.0141\;{\mathrm{mm}}^{-1}$ for $\mu s^{\prime }$.

The optical properties of the fabricated skin phantoms were compared with values reported in two *in vivo* studies, across the 750- to 900-nm wavelength range. Saager et al.^35^ measured the optical properties of human skin using spatial frequency domain spectroscopy and classified subjects according to the Fitzpatrick skin phototype scale. In this comparison, the pale, medium, and dark skin phantoms were compared with the values reported for Fitzpatrick II, IV, and VI, respectively. In addition, data from Tseng et al.,^36^ who measured optical properties using diffuse optical spectroscopy and grouped subjects according to ancestry, were used for comparison. In this case, the pale, medium, and dark phantoms were compared with the values reported for Caucasian, Asian, and African subjects, respectively.

**Fig. 14:**
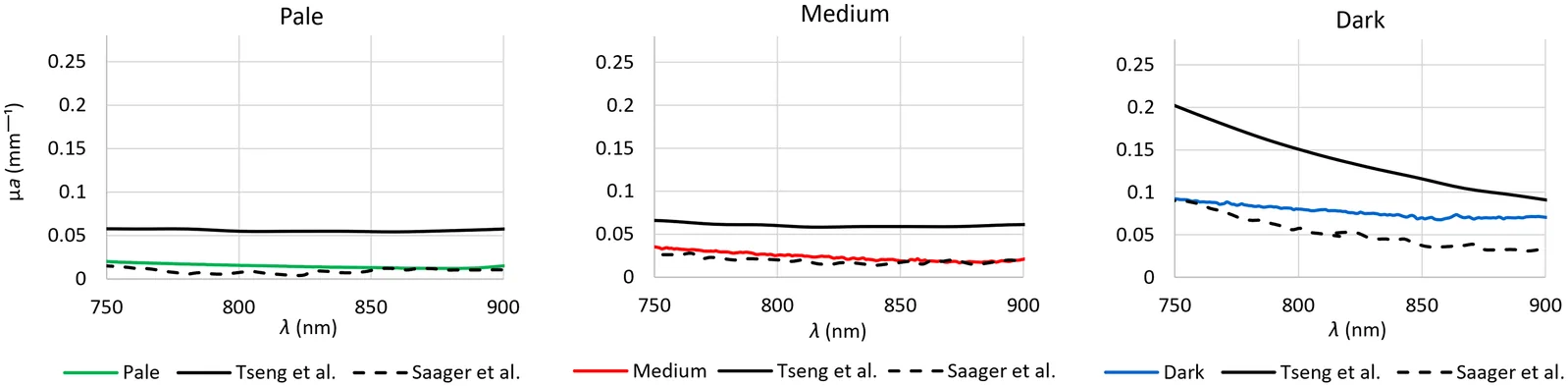
Absorption coefficient (μa) of the fabricated skin phantoms compared with *in vivo* measurements. Phantoms (pale, medium, and dark) are compared with *in vivo* ranges reported by Saager et al. (Fitzpatrick II, IV, and VI) and Tseng et al. (Caucasian, Asian, and African).

For all skin phantoms, the measured absorption coefficients fall within the range of values reported in the literature. A clear trend is observed in which the dark skin phantom exhibits higher absorption than the medium one, which also exhibits higher absorption than the pale phantom across all wavelengths. This behavior is consistent with *in vivo* observations, where increased skin pigmentation leads to higher optical absorption. Figure 14 shows the absorption coefficient (μa) of the three skin phantoms across the measured wavelength range, together with the ranges reported *in vivo*. The maximum standard deviation across the measured range was $0.0126\;{\mathrm{mm}}^{-1}$^.^

**Fig. 15:**
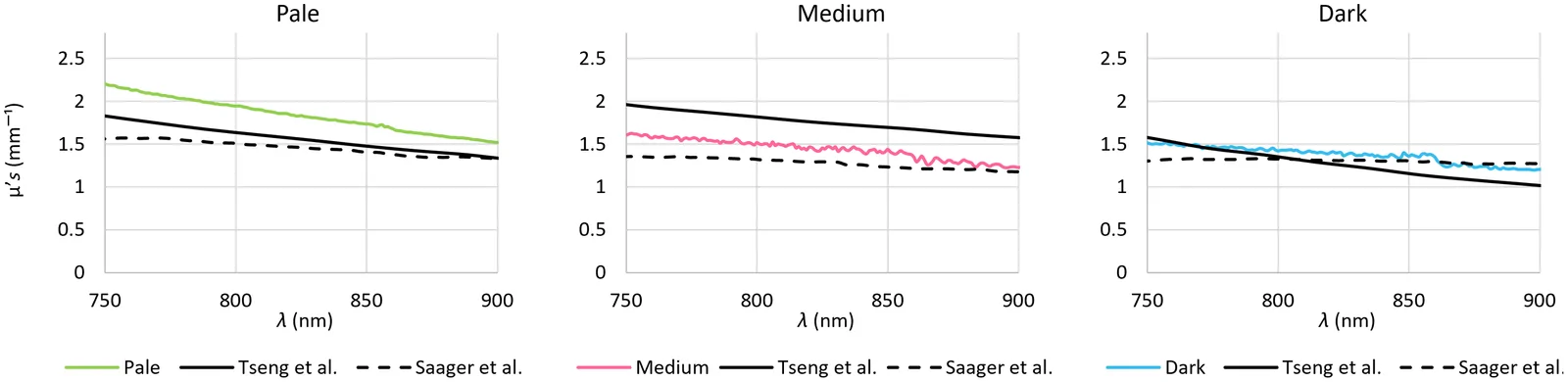
Reduced scattering coefficient ($\mu s^{\prime }$) of the fabricated skin phantoms compared with *in vivo* measurements. Phantoms (pale, medium, and dark) are compared with *in vivo* ranges reported by Saager et al. (Fitzpatrick II, IV, and VI) and Tseng et al. (Caucasian, Asian, and African).

In contrast to absorption, the literature does not report a consistent trend in the reduced scattering coefficient ($\mu s^{\prime }$) with skin pigmentation. This is expected because scattering in skin is primarily determined by structural components such as collagen fibers and cellular structures, which are less dependent on melanin concentration.^36^ Tseng et al.^36^ reported slightly higher $\mu s^{\prime }$ values for medium skin compared with other skin types, while Saager et al.^35^ observed similar $\mu s^{\prime }$ across pigmentation groups. The $\mu s^{\prime }$ values measured for all skin phantoms are shown in Fig. 15 and fall within the same order of magnitude as those reported *in vivo* and lie within or close to the reported ranges across the measured wavelengths. The maximum standard deviation across the measured range was $0.0445\;{\mathrm{mm}}^{-1}$.

It should be noted that the literature values used for comparison should not be interpreted as absolute reference standards. Many *in vivo* optical property studies are based on relatively small cohorts; for example, the measurements reported by Tseng et al.^36^ were obtained from only five volunteers per ancestry group. In addition, differences in measurement techniques and anatomical sites across studies can introduce variability in reported optical properties. Nevertheless, these data provide a useful reference for assessing general trends and order of magnitude when validating tissue-mimicking phantoms.

To assess optical stability over time, optical properties were also measured five months after fabrication. The scalp and skin phantom layers’ optical properties remained stable, consistent with previous reports of stability of incorporated optical absorbers using Platsil Gel-00.^37^ The optical stability of the blood-mimicking fluid was not as robust; therefore, a batch of the fluid was prepared less than 2 weeks before phantom PPG evaluation

Colorimetric and melanometric measurements of the fabricated skin phantoms were performed. The measured erythema index (*E*), melanin index (*M*), Individual Typology Angle (ITA°), and CIE L*a*b* parameters are summarized in Table 2.

**Table 2:** Colorimetric and melanometric measurements of the fabricated skin phantoms. Measurements were obtained using the Skin ColorCatch device. *E*, erythema index; *M*, melanin index; ITA°, Individual Typology Angle; L*, a*, b*, CIE color space parameters. The final column indicates the skin classification derived from ITA° according to the Skin ColorCatch manufacturer’s guidelines.

Skin	*E*	*M*	ITA°	L*	a*	b*	Device skin classification
Pale	472	544	24	64	9	32	“Tanned”
Medium	499	818	−24	39	13	25	“Brown”
Dark	486	850	−43	34	12	17	“Dark”

The ITA° showed a clear progression from positive values in the pale phantom to increasingly negative values in the medium and dark phantoms, consistent with trends reported in human skin.^30^^,^^31^ According to the Skin ColorCatch device classification based on ITA°, the fabricated phantoms correspond to the categories “tanned,” “brown,” and “dark.”

The L* values, representing lightness, ranged from 64 for the pale phantom to 34 for the dark phantom, following the expected decrease in brightness with increasing pigmentation and falling within the range reported for human skin in the literature.^30^^,^^31^ The a* values, which represent the red–green axis, showed a similar order of magnitude to those reported *in vivo* but did not follow a strictly monotonic trend across pigmentation levels. This behavior is consistent with observations in the human skin,^30^^,^^31^ where erythema-related variations can influence a* independently of pigmentation. The b* values, corresponding to the yellow–blue axis, also agree with typical values reported *in vivo*.^30^^,^^31^ These results confirm that the fabricated skin phantoms reproduce pigmentation-dependent optical and colorimetric characteristics consistent with human skin.

### 3.3 System Evaluation

**Fig. 16:**
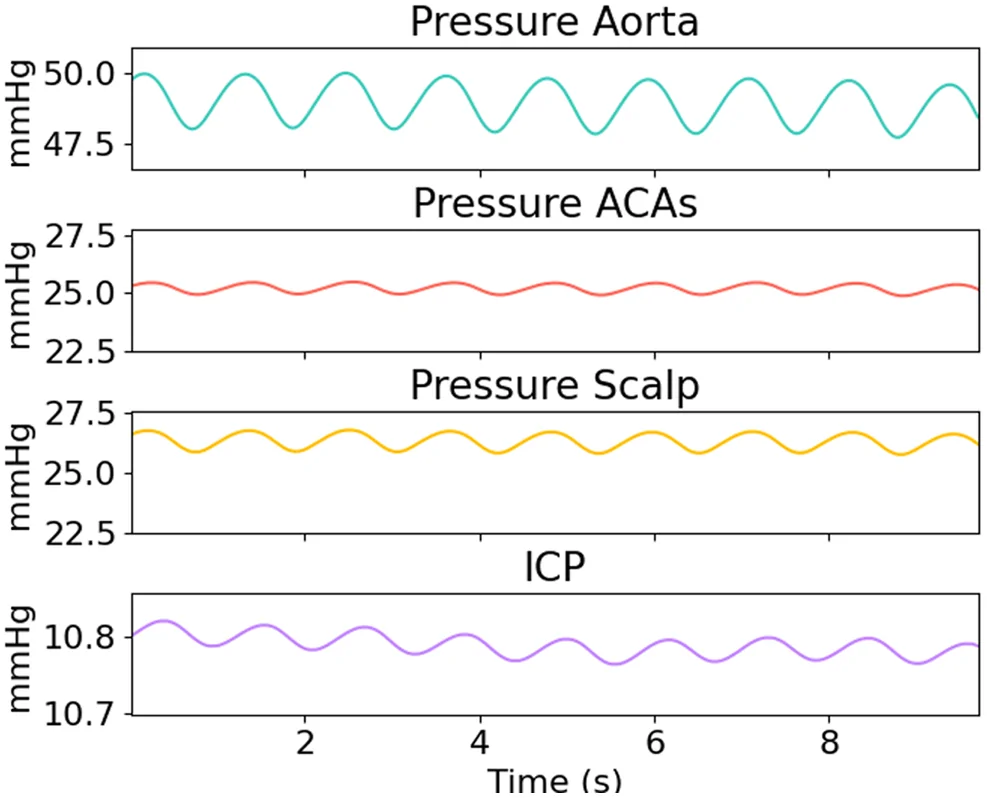
Pressure waveforms measured within the cardiovascular system. Representative 10-s time windows of pressure measured at the ascending aorta, ACAs inlet, scalp inlet, and ICP.

The hemodynamic behavior of the cardiovascular system was evaluated by monitoring pressures at four locations. Representative pressure waveforms are shown in Fig. 16 for the ascending aorta, ACA inlet, scalp inlet, and ICP. The pulsatile pump produced stable periodic pressure oscillations corresponding to the programmed heart rate of 60 beats/min. The mean pressures were ∼49  mmHg at the ascending aorta and 25 to 26 mmHg at both the ACAs and scalp inlets. ICP showed small pulsatile oscillations synchronized with the cardiac cycle. The results indicate that the cardiovascular system provides stable and physiologically consistent hemodynamic behavior suitable for optical sensor evaluation.

**Fig. 17:**
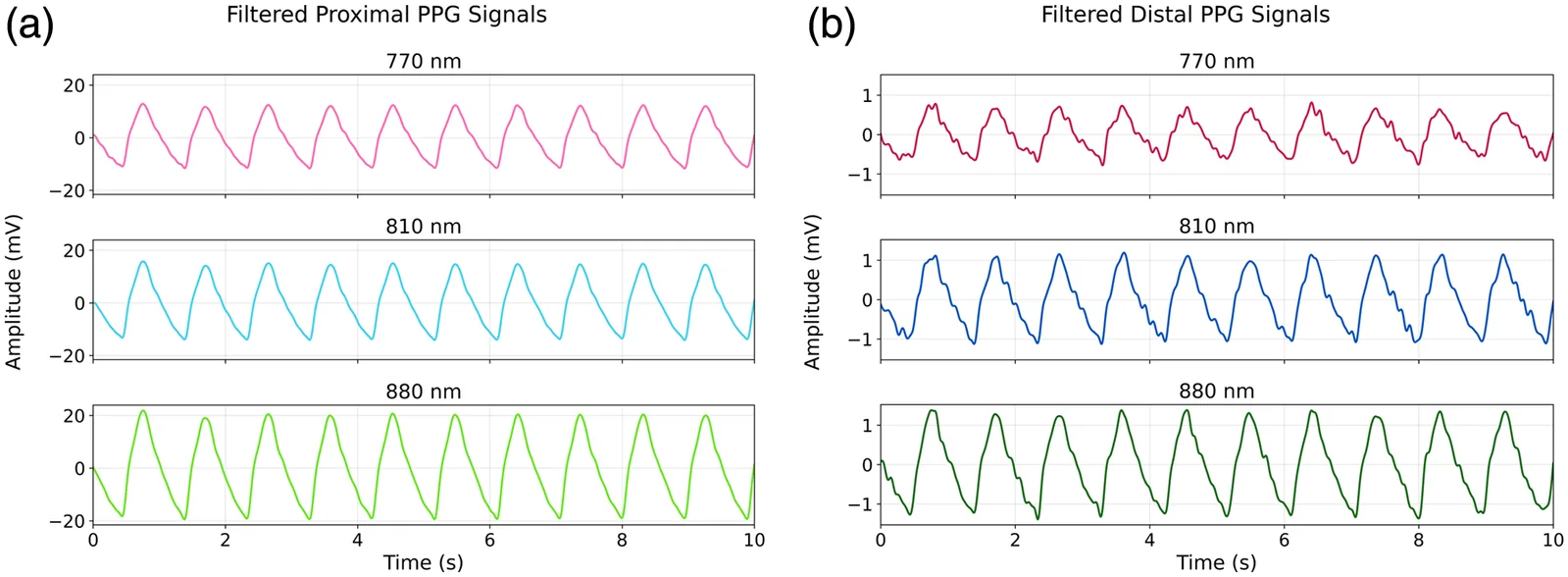
PPG signals acquired from the head phantom. (a) Signals measured at the proximal detector (1-cm source–detector separation) and (b) distal detector (3-cm separation). The signals correspond to a representative 10-s time window and were band-pass filtered (0.5 to 10 Hz). The *y*-axis scale is shared across wavelengths within each detector but differs between proximal and distal signals.

Cerebral PPG signals were acquired from the head phantom at three wavelengths (770, 810, and 880 nm) and at two source–detector separations (proximal: 1 cm; distal: 3 cm). The signals were then processed using a Butterworth band-pass filter (0.5 to 10 Hz, fourth order). Clear pulsatile waveforms corresponding to the programmed heart rate were observed across all wavelengths and detectors, as shown in Fig. 17. The distal detector exhibited lower signal amplitude compared with the proximal detector, consistent with increased photon path length and attenuation through the tissue layers. Amplitude slightly increased with wavelength for both photodetectors, with the largest amplitudes observed at 880 nm. These results demonstrate that the multilayer head phantom produces measurable pulsatile optical signals and can be used for controlled evaluation of cerebral PPG systems.

The developed head phantom also provides a versatile platform for investigating confounders in cerebral PPG monitoring. The integrated cardiovascular system enables independent control of perfusion in the brain and extracranial layer, allowing systematic evaluation of the contribution of extracranial circulation to cerebral PPG signals. In addition, the modular design of the phantom incorporates interchangeable skin layers representing different pigmentation levels, enabling controlled investigation of the impact of skin pigmentation on optical brain monitoring. Together, these characteristics allow the phantom to support reproducible experimental studies aimed at improving the interpretation and reliability of cerebral PPG monitoring.

## 4 Limitations and Future Work

The developed multilayer head phantom reproduces key anatomical and optical characteristics of the human head and provides a platform to acquire pulsatile optical signals. However, the phantom does not replicate all aspects of human physiology. The vascular geometry represents a simplified model of circulation and does not replicate the full complexity of the human vascular network. Physiological mechanisms such as cerebral autoregulation, active vascular responses, and dynamic changes in blood oxygenation were not reproduced. Inter-subject anatomical and optical variability is not represented, as the phantom incorporates fixed layer thicknesses for the skin, scalp, and skull, as well as fixed optical properties, which are known to vary across individuals. Skin pigmentation variability was also simplified to three representative pigmentation groups.

Future work will exploit the modular design of the phantom to investigate factors influencing cerebral PPG measurements. In particular, the system will enable controlled evaluation of the contribution of extracranial circulation to cerebral PPG signals, as well as investigation of the impact of skin pigmentation on optical measurements. Furthermore, blood analogs that allow controlled variation in oxygenation could be incorporated to study the combined effects of perfusion and blood oxygenation on cerebral PPG signals.

## 5 Conclusion

This study presented the design, fabrication, and evaluation of a multilayer head phantom replicating key anatomical structures of the human head. The phantom incorporates the brain, skull, scalp, and interchangeable skin layers, together with a cardiovascular system capable of generating controlled pulsatile flow. Optical characterization confirmed that the fabricated scalp and skin layers exhibit μa and $\mu s^{\prime }$ consistent with values reported for human tissues across the near-infrared wavelength range. In addition, colorimetric measurements demonstrated that the fabricated skin layers reproduce pigmentation-dependent characteristics representative of different skin tones. The integrated cardiovascular system enabled stable hemodynamic behavior and the generation of measurable pulsatile optical signals across multiple wavelengths and source–detector separations. Together, these results demonstrate that the developed phantom provides a realistic platform for experimental investigation of cerebral PPG signals and for controlled evaluation of factors that may influence optical brain monitoring.

## Data Availability

The data produced in this study are available upon reasonable request.
